# Sulfation of Glycosaminoglycans Modulates the Cell Cycle of Embryonic Mouse Spinal Cord Neural Stem Cells

**DOI:** 10.3389/fcell.2021.643060

**Published:** 2021-06-08

**Authors:** Elena Schaberg, Ursula Theocharidis, Marcus May, Katrin Lessmann, Timm Schroeder, Andreas Faissner

**Affiliations:** ^1^Department for Cell Morphology and Molecular Neurobiology, Ruhr University Bochum, Bochum, Germany; ^2^Department of Biosystems Science and Engineering, ETH Zürich, Zurich, Switzerland

**Keywords:** chondroitin sulfate proteoglycan, extracellular matrix, single-cell tracking, spinal cord, sodium chlorate, sulfation, stem cell niche

## Abstract

In the developing spinal cord neural stem and progenitor cells (NSPCs) secrete and are surrounded by extracellular matrix (ECM) molecules that influence their lineage decisions. The chondroitin sulfate proteoglycan (CSPG) DSD-1-PG is an isoform of receptor protein tyrosine phosphatase-beta/zeta (RPTPβ/ζ), a *trans-*membrane receptor expressed by NSPCs. The chondroitin sulfate glycosaminoglycan chains are sulfated at distinct positions by sulfotransferases, thereby generating the distinct DSD-1-epitope that is recognized by the monoclonal antibody (mAb) 473HD. We detected the epitope, the critical enzymes and RPTPβ/ζ in the developing spinal cord. To obtain insight into potential biological functions, we exposed spinal cord NSPCs to sodium chlorate. The reagent suppresses the sulfation of glycosaminoglycans, thereby erasing any sulfation code expressed by the glycosaminoglycan polymers. When NSPCs were treated with chlorate and cultivated in the presence of FGF2, their proliferation rate was clearly reduced, while NSPCs exposed to EGF were less affected. Time-lapse video microscopy and subsequent single-cell tracking revealed that pedigrees of NSPCs cultivated with FGF2 were strongly disrupted when sulfation was suppressed. Furthermore, the NSPCs displayed a protracted cell cycle length. We conclude that the inhibition of sulfation with sodium chlorate interferes with the FGF2-dependent cell cycle progression in spinal cord NSPCs.

## Introduction

The extracellular matrix (ECM) is a crucial determining structure in the developing central nervous system (CNS) that influences cell proliferation, lineage decisions and differentiation processes of neural stem and progenitor cells (NSPCs) (Barros et al., 2011; Theocharidis et al., 2014; Faissner and Reinhard, 2015). The ECM consists of glycoproteins, proteoglycans and complex glycans that either loosely pervade the pericellular space as interstitial ECM or assemble to complex interactomes, e.g., collagen fibrils or basal membranes. The ECM regulates a large range of cellular behaviors by activating distinct receptor systems, for example cell based heterodimeric integrin receptors (Barros et al., 2011). Based on bioinformatic analyses of the ECM constituents around 300 genes have been attributed to the core matrisome, about 35 of which encode proteoglycans (Hynes and Naba, 2012; Naba et al., 2012). The latter consist of a protein core and at least one covalently bound glycosaminoglycan (GAG) carbohydrate chain. Based on the type of glycan proteoglycans can be classified, e.g., in chondroitin sulfates (CSPG) and heparan sulfate proteoglycans (HSPGs) (Iozzo and Schaefer, 2015). The GAGs consist of long chains of carbohydrate dimers that are sulfated at distinct positions and thereby endowed with specific docking sites for proteins such as morphogens, cytokines or other ECM compounds (Purushothaman et al., 2012; Mikami and Kitagawa, 2017). Sulfated proteoglycans are expressed during CNS development, provide signaling cues and are involved in a variety of processes such as cell proliferation, differentiation, migration of neural progenitors or synaptogenesis (Iozzo and Schaefer, 2015; Maeda, 2015; Smith et al., 2015; Mikami and Kitagawa, 2017; Song and Dityatev, 2018).

Chondroitin sulfate GAGs (CS-GAGs) and heparan sulfate GAGs (HS-GAGs) are the two most commonly expressed GAGs in the developing CNS (Smith et al., 2015). Distinct sulfotransferases modify the GAGs in a spatially and temporally regulated manner and are expressed in neurogenic regions of the developing and adult brain and in neural stem cells in culture (Akita et al., 2008). Enzymatic digestion of CS-GAGs by chondroitinase ABC (ChABC) reduced proliferation and differentiation of cortical progenitors (von Holst et al., 2006; Sirko et al., 2007). The sulfation of GAG chains occurs by the transfer of sulfate groups from the donor 3′-phosphoadenosine 5′-phosphosulfate (PAPS). This enzymatic reaction can be competitively inhibited by sodium chlorate (NaClO_3_) (Sirko et al., 2010a). Sodium chlorate is a strong oxidant that has been used as herbicide until it was banned in the European Union in 2009 and is still applied as food supplement in live animal stocks in agriculture (Smith et al., 2012). We have shown previously that chlorate suppresses the sulfation of CS-GAGs in general, ablates the DSD-1-CS-epitope that is expressed by phosphacan and RPTP-β/ζ and thereby attenuates the proliferation of telencephalic NSPCs and spinal cord-derived neurospheres (Clement et al., 1998; Akita et al., 2008; Karus et al., 2012). In order to assess the potential functions of proteoglycans from the phosphacan/RPTP-β/ζ family we examined their expression and the presence of distinct CS-sulfotransferases during spinal cord development. To gain insight into the influence of sulfated GAGs on the cell cycle, we performed time-lapse video microscopy and single cell tracking of spinal cord progenitors treated with sodium chlorate, as a potent pharmacological sulfation inhibitor (Eilken et al., 2009; Rieger et al., 2009; Sirko et al., 2010a; Costa et al., 2011; Hoppe et al., 2016; May et al., 2018). Here, we show that the proliferation and lineage relationship of spinal cord-derived NSPCs cultivated in the presence of FGF2 are strongly altered by suppressing sulfation. This observation can be explained by an elongated cell cycle *in vitro*.

## Materials and Methods

All experiments conform to the relevant regulatory standards.

### Animals

Wild-type SV129 mice (*Mus musculus*) were used in accordance with the European Council Directive of September 22, 2010 (2010/63/EU) for care of laboratory animals and approved by the animal care committee of North Rhine-Westphalia, Germany, based at the LANUV (Landesamt für Umweltschutz, Naturschutz und Verbraucherschutz, Nordrhein-Westphalen, Recklinghausen, Germany). The study was supervised by the animal welfare commissioner of Ruhr-University. Male and female SV129 mice were housed individually with a constant 12-h light-dark cycle and access to food and water *ad libitum*. All efforts were made to reduce the number of animals in the experiments. For the experiments we used embryos of both sexes from time mated pregnant SV129 mice. The day of the vaginal plug was defined as embryonic day (E) 0.5 and the age of the embryos was verified by the determination of the Theiler stage. The experiments were performed with embryos at the age of embryonic day 15.5 (E15.5).

### Tissue Preparation for Spinal Cord Sections

E15.5 mouse embryos were decapitated and the tail was removed prior to an overnight incubation of the trunks in 4% (w/v) paraformaldehyde (PFA, Carl ROTH, Karlsruhe, Germany), dissolved in phosphate-buffered saline (PBS, 137 mM NaCl, 3 mM KCl, 6.5 mM Na_2_HOP_4_+2H_2_O, 1.5 mM KH_2_PO_4_, pH 7.3) at 4°C. The tissue was dehydrated in 20% (w/v) sucrose in DEPC-PBS (PBS treated with 0.1% (v/v) diethyl pyrocarbonate (DEPC, AppliChem, Darmstadt, Germany) over night before autoclaving) for 2 days before the embedding in Leica (Leica Biosystems, Richmond, IL, United States) tissue freezing medium. Horizontal cryosections with 14 μm thickness were cut in the lumbo-sacral region of the embryos at the Leica CM3050S cryostat and thaw-mounted on SuperFrost Plus glass slides (Menzel GmbH, Braunschweig, Germany). Animals from at least three different litters were used for the experiments and processed simultaneously. Figures show representative results from the independent experiments.

### Immunohistochemistry

A detailed list of primary antibodies can be found in Supplementary Table 2. Cryosections were rehydrated and blocked in PBS (including 1.7% (w/v) NaCl) + 10% (v/v) goat serum for 1 h before the antibodies βIII-tubulin (Sigma, 1:300), polyclonal anti-phosphacan (batch: KAF13[2], 1:300) and 473HD against the DSD-1-epitope (1:300; both (Faissner et al., 1994)) were applied in PBT1 [PBS with 1% (w/v) BSA (bovine serum albumin, ROTH) and 0.1% (v/v) triton-X100 (AppliChem)] with 5% (v/v) goat serum (Jackson ImmunoResearch). For the better accessibility of nuclear antigens the sections for the Islet-1/2 staining were boiled in 0.01 M citrate buffer for 10 min and then cooled down in the buffer for 5 min at room temperature and 10 min on ice. They were washed in 1× PBS before the application of the primary antibodies 473HD and Isl-1/2 (clone 39.4D5, DSHB; 1:200 hybridoma supernatant) in PBT-1. All primary antibodies were incubated for 2 h at room temperature and subsequently over night at 4°C and then washed twice with PBS. The secondary antibodies (α-rat-IgM Cy3, 1:600 and α-rabbit-AF488, 1:400, both from Jackson ImmunoResearch) were added in PBS/A (PBS with 0.1% (w/v) BSA) for 2 h at room temperature. To detect cell nuclei bisbenzimide (DAPI, Sigma) was included in the secondary antibody solution at a final concentration of 1:100,000. After washing twice with PBS, the sections were mounted with immumount (Shandon/Thermo Fisher Scientific) and analyzed by fluorescent microscopy at the AxioZoom V16 using ZEN 2009 pro Software (Carl Zeiss AG).

### Reverse Transcription Polymerase Chain Reaction (RT-PCR)

Spinal cord tissue from the lumbo-sacral part was isolated from E15.5 embryos and instantly frozen on dry ice. Total RNA was isolated from the tissue using the Sigma Mammalian GenElute-Total RNA miniprep Kit with an interposed DNase digestion step (On-Column DNaseI Digestion Set, Sigma). 1 μg of RNA was reverse-transcribed to cDNA using the first strand cDNA synthesis kit from Thermo Fisher Scientific adapted to a published protocol (von Holst et al., 2007; Sirko et al., 2010a). PCRs for the detection of RPTP-β/ζ /DSD-1-PG, the sulfotransferases and β-actin were performed using the primers and cycling conditions that can be found in Supplementary Table 3. All PCRs contained 1 μl cDNA, 5 pmol of each primer (synthesized by Sigma), 5 nmol dNTPs (Thermo Fisher Scientific), 10× reaction buffer and 1.25 U Taq-polymerase (both from Sigma) and were incubated in a Master Cycler Gradient (Eppendorf).

### *In situ* Hybridizations

Primers for the cloning of all used probes can be found in Supplementary Table 3. The probe detecting DSD-1-PG/RPTP-β/ζ was generated based on the cDNA sequence of the mouse *ptprz1* gene within the common part of the isoforms in the carbonic anhydrase domain and spacer region (Garwood et al., 1999). The sequence was amplified from embryonic mouse brain cDNA and cloned into the pCRII-TOPO vector (Thermo Fisher Scientific) according to manufacturer’s instructions. Vectors were isolated with the QIAprep Spin miniprep kit (QIAGEN) and cut with the restriction enzymes XhoI and HindIII (Thermo Fisher Scientific) for linearization. T7 and Sp6 RNA polymerases (Thermo Fisher Scientific) were used in combination with the DIG RNA labeling kit (Roche) to synthesize the sense and anti-sense riboprobes, respectively.

The sections for the *in situ* hybridizations were treated according to the protocol from Akita et al. (2008) with the aforementioned *ptprz1* riboprobes and the sulfotransferase probes listed in Supplementary Table 3. The cryosections were dried at room temperature, primed in 0.1 M TAE (triethanolamine, pH 8.0) and then acetylated with 0.25% (v/v) acetic anhydride in 0.1 M TAE for 10 min before two washing steps with 50 mM PB (phosphate buffer, NaH_2_PO_4_/Na_2_HPO_4_, pH 7.3). We performed a pre-incubation step in hybridization buffer (50% (v/v) formamide, 10% (w/v) dextran sulfate, 1× Denhardt’s reagent (Sigma), 100 μg ml^–1^ yeast RNA, 250 μg ml^–1^ salmon sperm DNA (Roche), 2× SSC (standard saline citrate, prepared as 20× SSC: 3 M NaCl, 0.3 M sodium citrate, pH 7.0), 50 mM sodium phosphate, pH 7.0, 0.2% (w/v) SDS) for 2 h at 60°C. In the meantime, the probes were denatured in 20 μl hybridization buffer (without SDS) for 5 min at 80°C before they were cooled on ice and supplemented with the respective amount of hybridization buffer to achieve the correct concentration and SDS. The hybridization was carried out over night at 60°C with probes against *ptprz1*, *Chst11*, *Ust*, *Chst3*, and *Chst7* and their respective sense control probes in a concentration of 1:100. On the next day the sections were stringently washed at 60°C in the following buffers: 4× SSC for 10 min, 2 × SSC containing 50% (v/v) formamide for 20 min twice, 2× SSC for 10 min, 0.2× SSC for 20 min twice, Tris–NaCl buffer (0.15 M NaCl, 0.1 M Tris–HCl, pH 7.5) for 10 min twice. Before the antibody was applied the sections were blocked in Tris–NaCl buffer containing 1% (w/v) skimmed milk powder. The anti-digoxigenin-AP coupled Fab fragments (Roche) were incubated at a concentration of 1:2000 in blocking buffer over night at 4°C on the sections. The sections were washed three times in Tris–NaCl buffer before the application of the alkaline phosphatase substrates nitroblue tetrazolium (NBT, 0.34 mg ml^–1^, Roche) and 5-bromo-4-chloro-3-indolyl phosphate (BCIP, 0.18 mg, Roche) in detection buffer containing 5% (w/v) polyvinyl alcohol, 0.1 M NaCl, 50 mM MgCl_2_, 0.1 M Tris–HCl, pH 9.5. The development of the color reaction was carried out at 37°C and stopped with 1 mM EDTA, 10 mM Tris–HCl, pH 7.5 when positive signals were visible as strong purple color under microscopic control. Sense controls were stopped simultaneously. The micrographs were taken with the AxioZoom V16 (Carl Zeiss AG) using ZEN 2009 pro software.

### Neurosphere Culture

The neurosphere culture system of spinal cord progenitors has been described (Karus et al., 2011). Dissection of the embryos for cell culture experiments was carried out in MEM (minimal essential medium, Sigma). The spinal cords of E15.5 old embryos were isolated and enzymatically digested with 30 U ml^–1^ Papain, 40 μg ml^–1^ DNaseI (both from Worthington) in the presence of 0.24 mg ml^–1^ L-Cysteine in MEM (Sigma). After centrifugation of the single cell suspension for 5 min at 1000 rpm the cell sediment was re-suspended in neurosphere medium consisting of DMEM/F-12 (1:1), 0.2 mg ml^–1^ L-glutamine (all from Sigma), 2% (v/v) B27, 100 U ml^–1^ penicillin, 100 μg ml^–1^ streptomycin (all from Invitrogen/Thermo Fisher Scientific), 20 ng ml^–1^ FGF2, 20 ng ml^–1^ EGF (both PeproTech) and 0.25 U ml^–1^ heparin (Sigma) for 6–7 days at 37°C and 6% (v/v) CO_2_ to get neurospheres (von Holst et al., 2006; Sirko et al., 2010a). For chlorate treatment we added 30 mM sodium chlorate (Sigma) pre-diluted in medium to the cultures as described previously (Akita et al., 2008; Sirko et al., 2010a; Karus et al., 2012).

### Single Cell Culture for Time-Lapse Video Microscopy

To prepare a single cell suspension the neurospheres were centrifuged for 5 min at 80 *g* and the resulting cell pellets were enzymatically digested with 0.05% (w/v) trypsin-EDTA in HBSS (Invitrogen/Thermo Fisher Scientific) for 5 min at 37°C. By adding 1 ml ovomucoid [1 mg ml^–1^ trypsin inhibitor (Sigma), 50 μg ml^–1^ BSA, 40 μg ml^–1^ DNaseI (Worthington) in L-15 medium (Sigma)] the digestion was stopped and after the mechanical dissociation the single cell suspension was centrifuged for 5 min at 80 *g*. Neurosphere medium was added and the cells were re-suspended. For time-lapse video microscopy 24 well plates (Thermo Fisher Scientific) were sequentially coated with 0.001% (v/v) poly-d-lysin (Sigma) in H_2_O, followed by 10 μg ml^–1^ laminin-1 (Invitrogen/Thermo Fisher Scientific) in PBS for 1 h at 37°C each. 30,000 cells/well were plated in neurosphere medium containing either 20 ng ml^–1^ EGF or 20 ng ml^–1^ FGF2 with 0.25 U ml^–1^ heparin (Sigma) and incubated at 37°C and 6% (v/v) CO_2_ for 4 days during the acquisition in order to perform time-lapse video microscopy. For chlorate treatment 30 mM sodium chlorate (Sigma) were added to the cultures. Each condition was performed in duplicates. The chlorate treated cells were cultivated and filmed simultaneously to the untreated cells on the same plate. At the same time, NSPCs isolated from the spinal cord of tenascin-C-deficient mice were analyzed in an analogous manner for an independent experimental approach. The dataset regarding the untreated control cells served as reference both for the chlorate-treated and the tenascin-C depleted NSPCs and has been published in the latter context (May et al., 2018). Here, as the experiments have been conducted in parallel, this data set of the control cells was used as reference to evaluate the influence of inhibited sulfation on spinal cord-derived NSPCs.

### Time Lapse Video Microscopy

The time lapse microscopy of spinal cord progenitors was performed at the Axiovert 200 M with the AxioCam HRm camera and a self-written VBA module remote (Eilken et al., 2009) controlling the Zeiss Axiovision program 4.8.2 (all Zeiss). Additionally, the devices Tempcontrol 37-2 digital and CTI-Controller 3700 digital (both PeCon) were used to create defined culture conditions with 37°C and 6% (v/v) CO_2_. Phase contrast images were taken every 5 min for at least 90 h. Five fields of view were defined for each well. Single cell tracking was performed using tTt, a computer program developed for the cell tracking and the generation of lineage trees on single cell level (Rieger et al., 2009; Hilsenbeck et al., 2016). Individual mother cells and their generated progeny were tracked over the cultivation period of 4 days. The duration between two cell divisions could be determined with high precision due to short intervals between the phase contrast images. Finally, full lineage trees originating from an individual stem cell could be constructed in that manner. A previous study of the laboratory illustrates this procedure, documenting the simultaneous construction of lineage trees in parallel with the time-lapse video (May et al., 2018). Movies were created using ImageJ 1.45r (National Institutes of Health) software and are played at a speed of five frames per second (see Supplementary Material). As cell culture dynamics could be monitored with high temporal resolution, quantification of proliferation and cell death events could be performed with ongoing cultivation. A detailed visualization of both processes has been published before (May et al., 2018).

### Data Analysis

To analyze the time-lapse video microscopy data the Kruskal–Wallis-Test with Dunn’s multiple comparisons test or the Mann–Whitney *U*-Test was used. A minimum of three independent experiments for each condition was performed. The data are illustrated as Box Whisker Plots with percentiles from 5 to 95%. All statistics and graphs were performed using GraphPad Prism^®^ software (Version 7, GraphPad Inc). *P*-values are given as ^∗^*P* ≤ 0.05, ^∗∗^*P* ≤ 0.1 and ^∗∗∗^*P* ≤ 0.001.

## Results

### Proteoglycans of the CS Type Are Present in the Developing Spinal Cord

**FIGURE 1:**
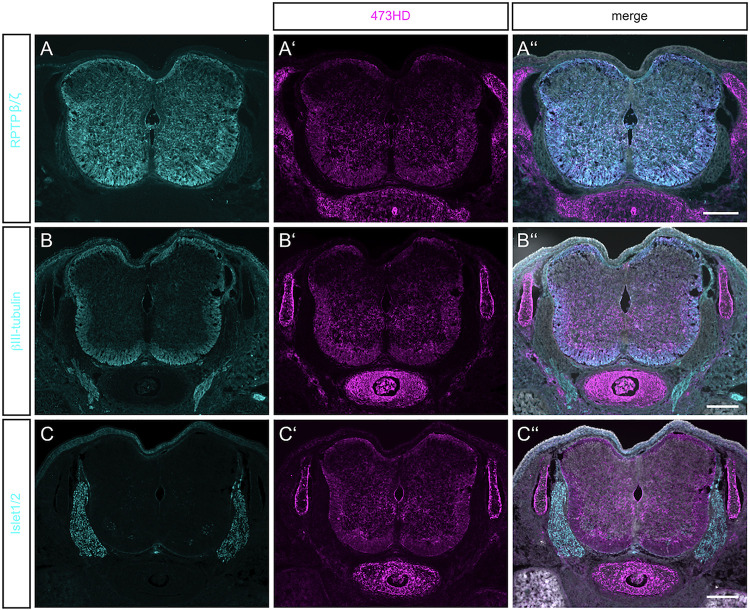
Detection of RPTPβ/ζ/DSD-1-PG in the embryonic spinal cord. **(A)** Immunohistochemistry with the polyclonal antibody anti-phosphacan revealed the expression of the CSPGs RPTP-β/ζ and DSD-1-PG/phosphacan in E15.5 mouse spinal cord. No labeling could be observed outside the CNS. **(A′)** The monoclonal antibody 473HD recognizes a specific carbohydrate motif on CSPGs that is present on RPTP-β/ζ /DSD-1-PG. Immuno-positive structures could also be found outside the CNS, especially in the underlying cartilage. **(A″)** The overlay of both labels and the nuclear DAPI staining (in white) showed a co-localization in the spinal cord, but solely 473HD-positive structures outside the CNS. **(B)** Young neurons in the developing spinal cord could be labeled with βIII-tubulin which is present especially in the ventral marginal zone. There, the expression partially overlapped with 473HD staining **(B′,B″)** **(C,C′,C″)**. The immune-positive signals for 473HD surrounded scattered Isl-1/2-positive nuclei in the ventral spinal cord but showed no reactivity in the dorsal root ganglia (DRG) where Isl-1/2-positive motoneuron nuclei were arranged. Scale bars: 200 μm.

In the neurogenic regions in the developing and adult brain CSPGs from the RPTP-β/ζ type can be found (Akita et al., 2008). The members of this family consist of a short and a long transmembrane receptor variant and the soluble CSPG phosphacan (Maurel et al., 1994), also described as the homolog DSD-1-PG in the mouse CNS (Faissner et al., 1994; Garwood et al., 1999). In order to obtain an overview about the expression of these CSPGs in the embryonic day 15.5 (E15.5) spinal cord immunohistochemistry was performed. We have previously shown that at this stage, subsequent to a period of intensive proliferation a phase of gliogenesis can be observed that is strongly modulated by the glycoprotein tenascin-C of the stem cell niche, indicating a significant role of the extracellular matrix (Karus et al., 2011; Faissner et al., 2017). The expression of RPTP-β/ζ was restricted to the CNS (Figure 1A) and could not be detected in any other non-neural tissues in horizontal sections of mouse embryos (data not shown). There was a clear expression in the spinal cord that was stronger in its ventral than in its dorsal half (Figure 1A). The proteins could be found in the intermediate as well as in the mantle regions.

The monoclonal antibody 473HD recognizes a specific subtype of GAG side chains and this motif is exposed by phosphacan/DSD-1-PG and the long isoform of the RPTP-β/ζ receptor (Faissner et al., 1994; Ito et al., 2005). Both contain a peptide sequence where GAG chains can be attached (Garwood et al., 1999). Its expression resembled the pattern seen for the RPTP-β/ζ staining, with a prominent overlay in the ventral part of the spinal cord (Figures 1A′,A″). The 473HD epitope was, however, not confined to the CNS but also clearly present in cartilage (see Figures 1A′–C′) and gut (data not shown). Thus, outside of the CNS the 473HD epitope seems to appear also on other proteoglycans beyond those recognized by the polyclonal RPTP-β/ζ antibodies (Ito et al., 2005). This is in accordance with an earlier study that reported expression of the DSD-1-epitope by Schwann cells in the sciatic nerve, in association with the CSPGs decorin and versican (Braunewell et al., 1995). 473HD expression overlapped with the signals obtained for βIII-tubulin immunostaining in the ventral mantle zone of the spinal cord, indicating a close association with motoneuron processes (Figures 1B′,B″). To have a closer look on this cell type we stained for the LIM homeobox transcription factors Islet-1 and -2 (Isl-1/2) (Figure 1C). The 473HD signal surrounded the large motoneuron cell bodies in the ventral basal plate (Figure 1C′) where single post-mitotic motoneuron nuclei were positive for Isl-1/2 (Figure 1C″). There was no overlap between 473HD and Isl-1/2 staining in the dorsal root ganglia (DRGs). 473HD was completely absent from the DRGs whereas the latter displayed clear expression of Isl-1/2. The association with motoneurons in the ventral spinal cord seemed to be concentrated to the stem and precursor cell stages in the ventral spinal cord as progenitors for post-mitotic motoneurons.

**FIGURE 2:**
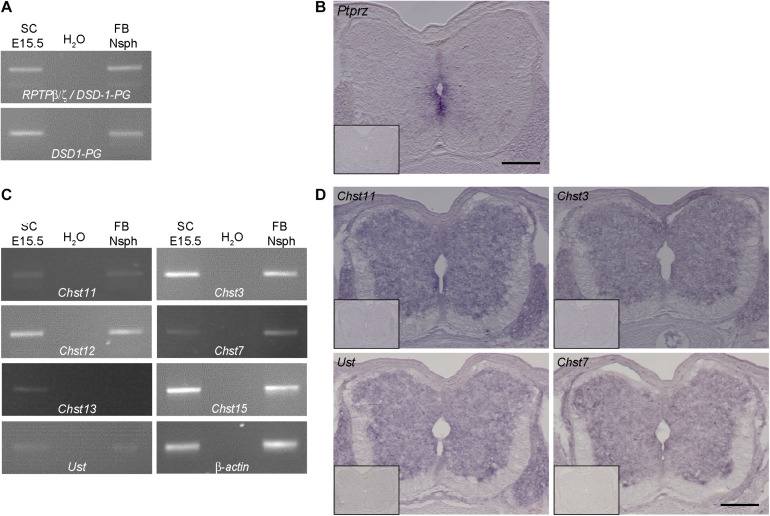
Expression analysis of RPTP-β/ζ /DSD-1-PG and CS-/DS-sulfotransferases in the developing spinal cord. **(A)** RT-PCR analysis revealed the presence of *DSD-1-PG/phosphacan* and RPTP-β/ζ mRNAs in tissue isolated from E15.5 spinal cords (E15.5 SC). The isoforms could be detected individually using primers located in the specific sequences of the mRNA (Garwood et al., 1999). **(B)** The mRNA of the *ptprz1* gene coding for a constant part of the RPTP-β/ζ and DSD-1 proteoglycan sequences was produced by cells surrounding the central canal, as could be seen by *in situ* hybridizations. Sections that were incubated with the sense riboprobe did not show any positive signals (insert). **(C)** *Chst11, Chst12, Chst13, Chst3*, and *Chst7* as well as *Chst15* and *Ust* genes could be detected in E15.5 spinal cord mRNA via RT-PCR. A representative spinal cord sample is shown together with the no-template control (H_2_O) and a positive control consisting of cDNA from neurospheres derived from embryonic brain tissue (Akita et al., 2008). Interestingly, although not present in telencephalic mRNA *Chst13* could be detected in E15.5 spinal cord tissue. *β-Actin* served as the reference gene. **(D)** Representative *in situ* hybridizations on E15.5 spinal cord sections showed positive signals for *Chst3, Chst7, Chst11, and Ust* in the central and intermediate zones of the spinal cord, but not in the marginal layer. Sulfotransferase message could be detected in the ventral as well as the dorsal spinal cord and the DRG. Sense controls (inserts) did not show detectable signals. Scale bars: 200 μm.

On the mRNA level all major isoforms of RPTP-β/ζ could be amplified by RT-PCR (Figure 2A upper panel), as well as the soluble DSD-1-PG (Figure 2A lower panel) indicating that both the long receptor and the secreted form were present and could represent core proteins of glycosylated proteoglycans. This approach did not, however, reveal the cellular source of the RPTP-β/ζ variants. Therefore, *in situ* hybridizations using a probe raised against the common constant part of DSD-1-PG/phosphacan and RPTP-β/ζ (Figure 2B) were performed. The proteoglycans were expressed in the spinal cord and produced by radial glia cells that surrounded the central canal. The signal was more intense in the ventral portion of the ventricular zone. This was consistent with previous findings that the 473HD epitope localizes with the nestin-positive neural stem/progenitor cells in the brain (von Holst et al., 2006) and spinal cord (Karus et al., 2012) and was more prominent in association with ventrally localized motoneurons than sensory neurons in the dorsal part. It has to be pointed out that DSD-1-PG/phosphacan is a secreted CSPG and thus can diffuse through the interstitial space of the developing spinal cord where it may be immobilized by selective receptor systems. For example, the transmembrane receptor RPTP-σ has been identified a CSPG receptor that is expressed by neurons and may convey inhibitory properties of CSPGs for axon outgrowth (Shen et al., 2009; Lang et al., 2015). Therefore, the distribution territory of the DSD-1-epitope on the one hand and the location of cells producing the core proteins do not necessarily overlap, which may explain different expression patterns.

### Sulfotransferases for CSPGs Are Expressed in the Spinal Cord

Previous investigations strongly suggested that DSD-1-PG/phosphacan and RPTP-β/ζ are the major, if not the sole core carrier proteins of the DSD-1-epitope in the CNS (Faissner et al., 1994; Schnadelbach et al., 1998; Garwood et al., 1999). The epitope contains the CSD unit that is sulfated at two positions (Clement et al., 1998; Sugahara et al., 2003). In a quest for enzymes that are required for the synthesis of this unit we next examined which CS-sulfotransferases are expressed in the developing spinal cord. We have previously detected several of these enzymes in the stem cell compartment of the developing and adult telencephalic neural stem cell niche (Akita et al., 2008). We focused on spinal cord tissue for the analysis of the expression of sulfotransferases that modulate the sulfation status of CSPGs. The reaction pathways catalyzed by these enzymes have been reviewed elsewhere (Sugahara and Mikami, 2007; Akita et al., 2008; Mikami and Kitagawa, 2017). We found different isoforms of the modifying enzymes at chondroitin 4-sulfate (Chst11, Chst12, and Chst13), chondroitin 6-sulfate (Chst3 and Chst7) as well as Chst15 and Ust via RT-PCR analysis in spinal cord tissue samples (Figure 2C). Interestingly, in the spinal cord we could detect the enzyme Chst13 that is not present in E13 brain tissue (Akita et al., 2008) or forebrain neurospheres that were used as positive control. Having established the expression of the mRNAs, it was of interest to specify the cellular sources of the sulfotransferase enzymes. Therefore, *in situ* hybridization was performed. Examining the mRNA distribution in the tissue by *in situ* hybridization the expression of the sulfotransferases *Chst11*, *Ust*, *Chst3*, and *Chst7* could be localized to the gray matter of the ventricular zone and the intermediate layer, but not in the white matter of the marginal layer (Figure 2D). Sulfotransferases could also clearly be detected in dorsal root ganglia, as visible on the sections hybridized with *Chst11*, *Chst3*, and *Chst7* probes (Figure 2D). These structures were neither positive for polyclonal phosphacan antibodies, nor the mAb 473HD (compare to Figure 1), which suggested that other proteoglycans are potential targets for sulfation by these enzymes.

### Proliferation and Cell Death Are Strongly Affected After Sodium Chlorate Treatment *in vitro*

**FIGURE 3:**
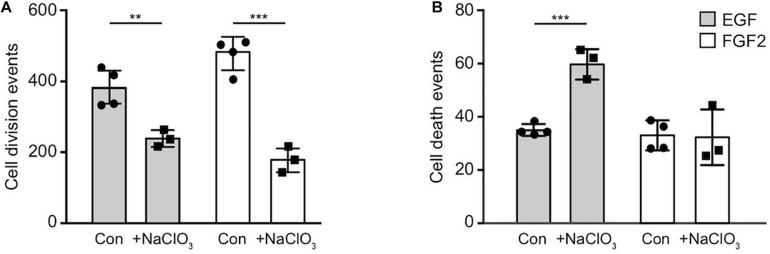
Inhibition of sulfation reduced the cell division both in response to EGF and FGF2 and increased cell death in an EGF-dependent manner. The total number of cell divisions and events of cell death was analyzed over a period of 2.5 days recorded by time-lapse video microscopy. **(A)** There was a strong reduction in the number of cell divisions of spinal cord progenitors exposed to either EGF or FGF2 caused by exposure to chlorate. **(B)** Cell death rates of progenitors appeared enhanced when treated with chlorate in the presence of EGF. Error bars indicate SD, **p ≤ 0.01, ***p ≤ 0.001 (*t*-test); *N* = 3.

Sulfated GAGs are essential for normal proliferation and cell cycle progression of spinal cord progenitors *in vitro* (Karus et al., 2012). The significance of those sulfation patterns can be tested by addition of sodium chlorate to cell cultures that competitively inhibits the synthesis of phosphoadenosine phosphosulfate (PAPS), the universal donor for sulfotransferases. Thereby, sodium chlorate blocks the sulfation of GAGs. This treatment efficiently reduces the expression level of the sulfation-dependent DSD-1-epitope (Clement et al., 1998; Karus et al., 2012). In order to gain deeper insight in the role of sulfation for the biology of NSPCs, we analyzed the total number of cell divisions and dying cells of control cultures without further addition of sodium chlorate and chlorate-treated (chlorate, NaClO_3_) progenitors over 2.5 days in phase contrast images obtained by time-lapse video microscopy (Figure 3). We noted a 2–3 fold reduction in cell divisions of chlorate-treated progenitors compared to control cultivated in the presence of either EGF or FGF2 (EGF, con: 382 ± 54, EGF+chlorate: 242 ± 36; *p* < 0.009; con *N* = 4, EGF+chlorate *N* = 3; FGF2, con: 477 ± 57, FGF2+chlorate: 182 ± 36; *p* < 0.001; con *N* = 4, FGF2+chlorate *N* = 3) (Figure 3A). Interestingly, the rate of cell death of spinal cord progenitors maintained in EGF was increased upon sodium chlorate treatment in comparison to the control, whereas progenitors kept in FGF2 and chlorate were barely affected in their survival (EGF, con: 35 ± 2, EGF+chlorate: 59 ± 6; *p* < 0.001 con *N* = 4, EGF+chlorate *N* = 3; FGF2, con: 33 ± 6, FGF2+chlorate: 32 ± 7; con *N* = 4, FGF2+chlorate *N* = 3) (Figure 3B). These results reveal the importance of sulfated GAGs for cell division and cell survival of spinal cord progenitors.

### Time-Lapse Video Microscopy Gives Insight Into Cell Cycle Behavior of Spinal Cord Progenitors

**FIGURE 4:**
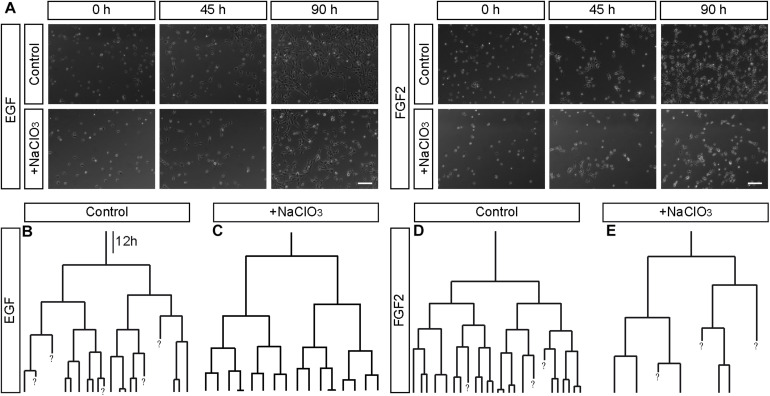
Representative phase contrast images and lineage trees of spinal cord progenitors *in vitro*. **(A)** Phase contrast images obtained by time-lapse video microscopy at 0, 45, and 90 h of untreated (control, con) and chlorate-treated (NaClO_3_) spinal cord progenitors exposed to EGF or FGF2. Untreated progenitors displayed higher cell densities after 90 h of cultivation than chlorate-treated cells. Additionally to the phase contrast images, exemplary movie sequences of all four conditions are included (Supplementary Movies 1–4). **(B–E)** Representative lineage trees of control and chlorate treated progenitors tracked in the presence of EGF **(B,C)** or FGF2 **(D,E)** are shown. There were some apparent differences between untreated control cells **(B,D)** and those exposed to inhibition of sulfation [NaClO_3_, **(C,E)**]. The cell divisions of most sibling-cells occurred in a highly synchronous way. Chlorate-treated cells in the presence of FGF2 divided asynchronously in comparison to control cells and produced strongly distorted lineage trees. A question mark “?” indicates cells which were not traceable any further. Scale bar: 100 μm. The dataset of the control has been used in a previous study conducted in parallel (May et al., 2018).

Sulfation of spinal cord progenitors is important for normal cell cycle progression, because inhibition of sulfation maintained more cells in the G2-phase and less on the M-phase of the cell cycle at embryonic stage E12.5, a phase of intense proliferation (Karus et al., 2012). With the aim to clarify the role of sulfated GAGs for the cell cycle in more detail, time-lapse video microscopy and single cell tracking of E15.5 spinal cord progenitors was performed. In this setting, cell divisions can be conveniently spotted and counted on the culture substrate, as reported previously (May et al., 2018). Thereby, the proliferative activity in the culture could be estimated. Dying cells could be identified as the cell bodies shrunk and eventually disappeared from the culture substrate. We compared untreated with chlorate treated progenitors exposed to EGF and FGF2. After 90 h, control progenitors proliferated and showed a higher cell density under both EGF (Supplementary Movie 1) and FGF2 (Supplementary Movie 3) conditions (Figure 4A). In contrast, the cell density visible after chlorate treatment was lower (Figure 4A). The focus of the current investigation was on the proliferation behavior of progenitors. Therefore, individual dividing cells were traced through several generations and their pedigrees constructed. Representative lineage trees of control progenitors showed synchronous cell cycles with a symmetric division mode of sibling cells exposed to EGF and FGF2 (Figures 4B,D, respectively). This phenomenon could also be observed for progenitors cultivated with chlorate and EGF (Figure 4C and Supplementary Movie 2). In contrast, chlorate-treated progenitors in the presence of FGF2 displayed a markedly different behavior (Supplementary Movie 4). The division mode appeared asymmetric and the sibling cells divided less synchronously (Figure 4E). Quantification of the cell cycle length illustrated that the mitogen FGF2 accelerated the cell cycle of normally sulfated spinal cord progenitors in the 2nd, 3rd, and 4th generation in comparison with EGF [compare to May et al. (2018)].

### Sodium Chlorate Interfered With the Cell Cycle of Progenitors Exposed to FGF2

**FIGURE 5:**
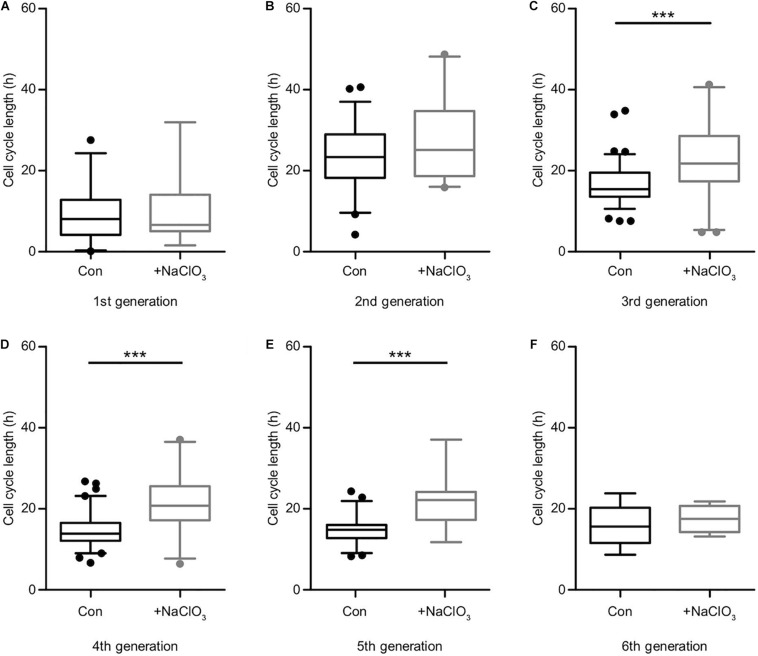
Suppression of sulfation lengthened the cell cycle in the presence of FGF2. Cell cycle lengths of control (black boxes) versus chlorate-treated (gray boxes) spinal cord progenitors maintained in the presence of FGF2 were compared. The cells from the first generation **(A)** were captured at different starting points of their division cycle and could only be tracked for an undefined portion of the running cell cycle. From the second generation **(B)** on the cell cycle lengths could be compared statistically. The inhibition of sulfation led to a slower cell division of progenitors treated with chlorate. The prolongation of the cell cycle was particularly striking between the 3rd up to the 5th round of division **(C–E)**. The sixth generation **(F)** was only traceable for a few cells (compare to the lineage tree in Figure 4E) ***p ≤ 0.001 (Mann–Whitney *U*-test); con: *N* = 4; chlorate: *N* = 3.

Comparing the different generations of every single condition helped to derive an estimate for the cell cycle length (Supplementary Table 1). It should be kept in mind that the data of the first generation were not precisely circumscribed, because the cell cycle state of the initially tracked mother cell was unknown when the tracking began. Therefore, the depicted time frames appear shorter than in the subsequent generations. In the second generation, the cell cycle length was the longest of all conditions, decreased with ongoing generation, and finally reached equivalence between control and treatment condition (Figure 5 and Supplementary Table 1). However, the inhibition of sulfation in the presence of FGF2 notably changed the behavior of the progenitors and led to an elongated cell cycle length in all generations (Figure 5 and Supplementary Table 1). Untreated control cells were compared with chlorate-treated cells in every single generation. We could observe a particularly strong difference concerning the cell cycle length after inhibition of sulfation in the 3rd, 4th, and 5th generation (Supplementary Table 1, Figures 5C–E; 3rd generation, con: 15.4 h vs. chlorate: 21.8 h; 4th generation, con: 13.9 h vs. chlorate: 20.8 h; 5th generation, con: 14.8 h vs. chlorate 22.2 h; control *N* = 4, chlorate *N* = 3). We conclude that sulfated GAGs are essential for normal cell cycle progression driven by FGF2.

### Inhibition of Sulfation Does Not Interfere With EGF Signaling

**FIGURE 6:**
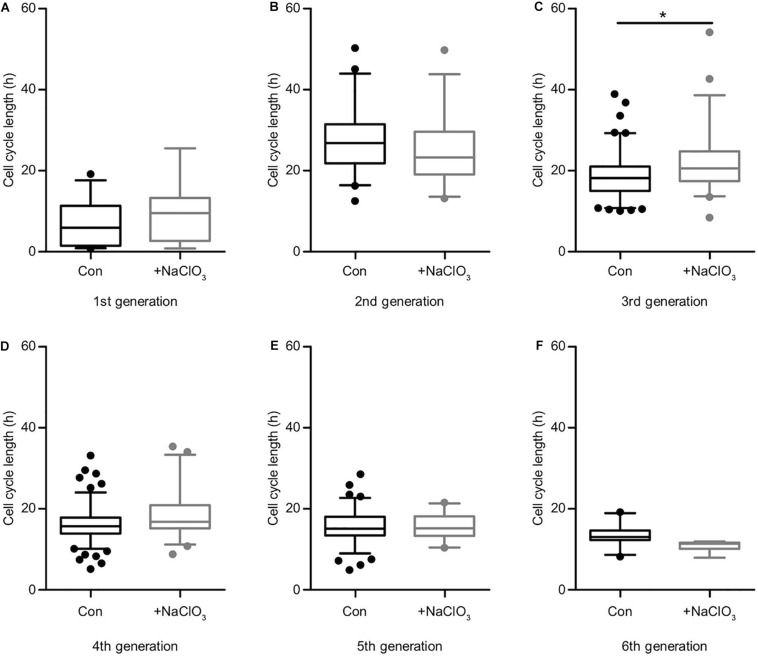
Sulfation of spinal cord progenitors had a minor influence on the cell cycle in the presence of EGF. The cell cycle lengths of control (black boxes) and chlorate-treated (gray boxes) spinal cord progenitors cultivated in the presence of EGF are shown for the first **(A)**, second **(B)**, third **(C)**, fourth **(D)**, fifth **(E)** and the sixth generation **(F)**. In the third generation **(C)** the inhibition of sulfation led to a slightly slower cell division compared to untreated cells. In all other generations, the suppression of sulfation had no significant influence on the cell cycle length. *p ≤ 0.05 (Mann-Whitney *U*-test); control: *N* = 4; chlorate: *N* = 3.

The analysis of the impact of sodium chlorate treatment of progenitors cultivated in the presence of EGF yielded a different result. Apart from generation 3 (Supplementary Table 1, Figure 6C; con: 18.2 h vs. chlorate: 20.6 h), where chlorate-treated cells divided more slowly than in the control condition, all other generations did not display any significant differences regarding cell cycle length (Supplementary Table 1 and Figures 6A–F). In the light of this result, we conclude that the inhibition of sulfation led to a significant reduction in cell division events and a significant increase of cell death in the presence of EGF (Figures 3A,B), but did not affect the lineage progression in spinal cord progenitors.

## Discussion

It is known HS-GAGs are necessary for normal FGF2 signaling and therefore HS-GAGs have an indirect influence on proliferation and differentiation (Eswarakumar et al., 2005; Matsuo and Kimura-Yoshida, 2013; Woodbury and Ikezu, 2014). Sulfated GAGs of proteoglycans play an important role in numerous processes during development and in adulthood (Iozzo and Schaefer, 2015; Wiese and Faissner, 2015; Mikami and Kitagawa, 2017). CS-GAGs are strongly expressed in stem cell niches of the CNS and regulate proliferation and differentiation of progenitors both in the cortex and spinal cord (Engel et al., 1996; Karus et al., 2012; Song and Dityatev, 2018). It has been shown that the DSD-1-epitope, a unique CS-motif, is expressed in the developing spinal cord and on radial glia cells of the cortex. The specific monoclonal antibody 473HD that recognizes the DSD-1-epitope reduces neurosphere formation when added to NSPC cultures, which reflects a functional role of the epitope in the stem cell niche (von Holst et al., 2006; Karus et al., 2012). The enzymatic digestion of specific CS-motifs by ChABC *in vivo* and *in vitro* decreases proliferation and self-renewal of telencephalic progenitors. Furthermore, this treatment leads to an increased number of differentiated astrocytes at the expense of neurons in a neurosphere differentiation assay (Sirko et al., 2007, 2010b). In contrast, spinal cord progenitors generate more immature neurons after inhibition of sulfation *in vitro* (Karus et al., 2012).

The proteoglycans of the RPTP-β/ζ family with its soluble member DSD-1-PG, the mouse homolog of phosphacan (Garwood et al., 1999; Faissner et al., 2006) were detected in the E15.5 spinal cord. Its presence in the close vicinity to neural stem and progenitor cells has been observed before in other CNS regions and developmental stages (von Holst et al., 2006; Sirko et al., 2007, 2010b; Akita et al., 2008; Klausmeyer et al., 2011; Karus et al., 2012), but whether these cells are indeed the source for these proteoglycans was unknown. We confirmed this by *in situ* hybridizations for the determining gene construct of *ptprz1* detecting the mRNAs of the RPTP-β/ζ transmembrane receptors and DSD-1-PG/phosphacan, the proteoglycan isoform derived therefrom. The signals surrounded the central canal of the spinal cord where the cell bodies of the progenitors are located. Immunohistochemistry with antibodies revealed the distribution of the molecules along the radial glia cell fibers stretching from the central canal to the surface of the spinal cord. The distribution of RPTP-β/ζ isoforms follows a gradient, with a more pronounced expression toward the ventral half of the spinal cord, where the motoneurons are located (Wiese and Faissner, 2015). Interestingly, motoneuron axons are guided by RPTP-β/ζ variants *in vitro* (Klausmeyer et al., 2011). The differential distribution may reflect diffusion of the proteoglycan and immobilization by specific receptor systems or an intrinsic difference of dorsal versus ventrally located NSPCs. We have reported that astrocyte progenitors assemble in the ventral half of the spinal cord at E15.5 (Karus et al., 2011). Significant heterogeneity of astrocytes has been concluded from transcriptome studies and bioinformatic analysis (John Lin et al., 2017; Fu et al., 2021). However, tools to enrich and separately study these asserted subpopulations in the spinal cord still remain to be developed.

Although the proteoglycans of the RPTP-β/ζ family are restricted to the CNS, the CS-GAG chains associated with these proteoglycans are widely distributed. Thus, the particular DSD-1 carbohydrate structure that is recognized by the mAb 473HD is attached to the long receptor form of RPTP-β/ζ and to DSD-1-PG, which appear to be the major, if not exclusive core carrier proteins in the central nervous system (Faissner et al., 1994; Garwood et al., 1999). However, immunohistochemistry detected the epitope also in cartilage where CSPGs such as aggrecan, decorin and biglycan are prominent, as well as the HSPGs perlecan, the syndecans and glypicans (Knudson and Knudson, 2001). The strong interaction of the 473HD antibody with the CS-domain D from shark cartilage has been shown in a detailed epitope characterization (Ito et al., 2005). An association with versican and decorin in the human sciatic nerve has been previously inferred (Braunewell et al., 1995). Thus, the DSD-1-epitope is presumably exposed by other core proteins in embryonic mesenchyme derived tissues.

The CS/DS-proteoglycan side chains are modified by sulfotransferases that add sulfate groups to the GAGs at 2-S, 4-S, or 6-S positions, which generates docking sites for a variety of proteins (Sugahara and Mikami, 2007; Purushothaman et al., 2012). A family of specialized enzymes creates a production line that modifies the glycan chains and restructures the CS-GAG structure and charge. We could detect the mRNA expression of diverse sulfotransferases that modify CSPGs (Akita et al., 2008; Mikami and Kitagawa, 2017) and found that the enzyme Chst13 is exclusively present in the spinal cord, but not in the forebrain neurosphere mRNA that we used as control. This is consistent with previous results where Chst13 could not be detected in forebrain tissue (Akita et al., 2008). The analysis of the four sulfotransferases that we analyzed by *in situ* hybridizations revealed expression in the cellular compartment of the developing spinal cord and in the adjacent tissues of the dorsal root ganglia. The enzymes modify GAG chains of the CSPG and DSPG types of which different members control cellular events in diverse tissues and are not clearly restricted to the central nervous system. Keratinocytes for example show hyperproliferation in a knockout model of Chst3 (Kitazawa et al., 2021). In contrast, the overexpression of another sulfotransferase, Chst15, is associated with tumor growth in pancreatic cancer (Matsuda et al., 2019). Interestingly, several reports have highlighted a significant role of sulfotransferases in the context of human pathophysiology, e.g., in the connective tissue (Mizumoto et al., 2013). As the DSD-1-epitope comprises the CS-D-A-D motif, at least the three distinct sulfotransferases are required for the biosynthesis of the A-, C-, and D-unit (Ito et al., 2005; Miyata and Kitagawa, 2017). The analysis of their respective roles for NSPC proliferation represents a challenging task for future studies.

Although proteoglycans are involved in cell proliferation little is known about the influence of the sulfated GAGs on the cell cycle itself. That is why we focused on the analysis of the cell cycle length of spinal cord progenitors by the use of time-lapse video microscopy and single cell tracking. The cell division events of progenitors treated with sodium chlorate were dramatically reduced compared to untreated progenitors. Inhibition of sulfation intensively interfered with FGF2-dependent cell cycle progression and altered the division mode. Generally, progenitors exposed to FGF2 divided faster compared with EGF treated progenitors, but after inhibition of the sulfation we observed the opposite effect. NSPCs from the embryonic spinal cord only sparsely display mitotic events without growth factor treatment (data not shown). It is assumed that the addition of growth factors to the medium overall reduces the cell cycle length. This has in fact been confirmed for adult neural stem cells of the subventricular zone, which are able to proliferate *in vitro* also in the absence of any exogenously supplied factors (Costa et al., 2011).

Sodium chlorate inhibits the synthesis of the universal sulfate donor PAPS and thereby also interferes with the sulfation of proteins (Baeuerle and Huttner, 1986). Sulfation of proteins is operated by tyrosylprotein sulfotransferases (TPST1 and TPST2 enzymes) and occurs at free tyrosine residues (Beisswanger et al., 1998). It has been reported for a number of proteins, where it may contribute to sorting and protein interactions. The biological functions of protein tyrosine sulfation are presently not well understood and under ongoing investigation (Yang et al., 2015). However, there is general agreement that sodium chlorate treatment is not toxic for animal cells in culture (Baeuerle and Huttner, 1986; Clement et al., 1998). Along these lines, as the treatment of neural cells with sodium chlorate also does not reduce the expression levels of the carrier core proteins DSD-1PG/phosphacan and RPTP-β/ζ (Akita et al., 2008), we are convinced that the major impact of sodium chlorate in our studies targeted the GAG-compartment.

Heparan sulfate GAGs are expressed in the subventricular zone of the CNS and are involved in the FGF2 signaling pathway (Lamanna et al., 2008; Yamaguchi et al., 2010; Matsuo and Kimura-Yoshida, 2013; Ravikumar et al., 2020). Interference with FGF2 signaling by the use of sodium chlorate led to a longer cell cycle length of spinal cord progenitors. This is in line with previous findings where less neurospheres were generated after inhibition of sulfation and exposure to FGF2 (Karus et al., 2012). The FGF2 signaling pathway is disrupted, because FGF2 needs a specific HS-GAG-motif to bind the FGF-receptor (Bowman and Bertozzi, 1999). In contrast, EGF does not bind to HSPGs (Higashiyama et al., 1993) and accordingly, EGF-dependent cell cycling is not critically affected by chlorate treatment in our experiments. CS-GAGs also play a role, because treatment of telencephalic NSPCs with ChABC reduced proliferation in response to FGF2, but not to EGF (Sirko et al., 2010b). Although we could measure only a minor influence of EGF on the cell cycle length of progenitors after sodium chlorate treatment, the total number of cell divisions was reduced. This effect was even more severe when the cells where exposed to FGF2. However, the rate of cell death in response to chlorate exposure was only increased in NSPCs cultivated in the presence of EGF. This was surprising, because it has been reported that sodium chlorate is not toxic for cells (Baeuerle and Huttner, 1986; Karus et al., 2012). On the other hand, chlorate treatment results in a relative accumulation of cells in the G2-phase of the cell cycle, which predisposes for cell death (Rieder, 2011; Karus et al., 2012). In agreement with this finding, NSPCs with suppressed sulfation displayed a longer cell cycle. Interestingly, HS-GAGs that are also targeted by chlorate are required to leave the self-renewal mode and switch to differentiation (Kraushaar et al., 2010).

In summary, we provide evidence that sulfated proteoglycans have an impact on the cell cycle of spinal cord progenitors. Different from approaches that were based on the degradation of CS-GAGs by chondroitinases we focused on the sulfation of the GAG-chains and demonstrate that these exert intrinsic effects on their own, independently of the core protein. Thereby, the results of the present study extend previous findings and illustrate the heterogeneity of the functions of GAGs and the importance of specific sulfation patterns for EGF and notably FGF2 related signaling and cell cycle progression.

## Data Availability Statement

The raw data supporting the conclusions of this article will be made available by the authors, without undue reservation.

## Ethics Statement

Wild-type SV129 mice (*Mus musculus*) were used in accordance with the European Council Directive of September 22, 2010 (2010/63/EU) for care of laboratory animals and approved by the animal care committee of North Rhine-Westphalia, Germany, based at the LANUV (Landesamt für Umweltschutz, Naturschutz und Verbraucherschutz, Nordrhein-Westphalen, Recklinghausen, Germany). The study was supervised by the animal welfare commissioner of Ruhr-University.

## Author Contributions

ES performed the experiments, analyzed the data, wrote the manuscript, and prepared the figures. UT developed the experimental design, performed the experiments, analyzed the data, and wrote the manuscript. MM performed the video microscopy experiments, analyzed the data, and wrote the manuscript. KL performed the video microscopy experiments. TS developed the video microscopy analysis and provided the software. AF developed the experimental design, supervised the work, revised the manuscript, and funded the study. All authors have read and approved the manuscript.

## Conflict of Interest

The authors declare that the research was conducted in the absence of any commercial or financial relationships that could be construed as a potential conflict of interest.
