# Supplementary material for: Sulfation of Glycosaminoglycans Modulates the Cell Cycle of Embryonic Mouse Spinal Cord Neural Stem Cells
Source: Front Cell Dev Biol. 2021 Jun 8;9:643060. doi: 10.3389/fcell.2021.643060 (PMC8217649; doi:10.3389/fcell.2021.643060)
Supplement: Supplementary file 1 [file Table_1.DOCX]

Sulfation of glycosaminoglycans modulates the cell cycle of embryonic mouse spinal cord neural stem cells

Elena Schaberg^1^, Ursula Theocharidis^1^, Marcus May^1^, Katrin Lessmann^1^, Timm Schroeder^2^, Andreas Faissner^1,*^

**Supplementary Data**

**Table S1**

| Generation | Median of cell cycle length in h (numbers of tracked cells) | | | |
| --- | --- | --- | --- | --- |
|  | FGF2 | | EGF | |
|  | control (taken from May et al. 2018) | NaClO_3_-treated | control (taken from May et al. 2018) | NaClO_3_-treated |
|  |  |  |  |  |
| 2 | 23,1 (n=55) | 24,8 (n=29) | 26,8 (n=57) | 23,3 (n=32) |
| 3 | 15,4 (n=93) | 21,8 (n=44) | 18,2 (n=100) | 20,6 (n=47) |
| 4 | 13,9 (n=80) | 20,8 (n=33) | 16,1 (n=140) | 17,2 (n=47) |
| 5 | 14,8 (n=48) | 22,2 (n=13) | 15,5 (n=94) | 15,6 (n=30) |
| 6 | 15,6 (n=4) | 17,5 (n=2) | 13,4 (n=26) | 11,8 (n=7) |

Median of the cell cycle length of control and chlorate-treated progenitors exposed to EGF and FGF2 obtained by time-lapse video microscopy and cell tracking is shown. The cell cycle length of control progenitors declined with increasing generation. The same was observed for chlorate-treated progenitors cultivated with EGF. But the inhibition of sulfation led to a milder reduction of the cell cycle length. Data analysis was performed with the Mann-Whitney U-Test of N=4 (control) and N=3 (chlorate) independently conducted experiments. Numbers of individually tracked cells (n) are shown in brackets.

**Table S2**: Antibodies used for immunohistochemistry

| **antigen** | **antibody** | **type** | **species** | **dilution IHC** | **reference/ manufacturer** | **order number / LOT** | **RRID** |  |
| --- | --- | --- | --- | --- | --- | --- | --- | --- |
| DSD-1-PG/ RPTPβ/ζ | KAF13[2] | pAB | rabbit | 1:300 | Faissner et al. 1994 | N/A | N/A |  |
| 473HD epitope | 473HD | IgM | rat | 1:300 | Faissner et al. 1994 | N/A | N/A |  |
| βIII-tubulin | βIII -tubulin (SDL.3D10) | IgG2b | mouse | 1:300 | Sigma | T8660  Lot 097M4835V | AB_477590 |  |
| Islet-1/2 | 39.4D5 | IgG2b | mouse | 1:200 | Developmental Studies Hybridoma Bank (DSHB), Jessell, T.M. / Brenner-Morton, S. | 39.4D5  (Purified supernatant, by Dr. Wiese) | AB_2314683 |  |
| **antibody** | **Type** | **Conjugate** | **Species** | **Dilution IHC** | **Manufacturer** | **Order number / Lot** | **RRID** | |
| Anti-rabbit | IgG | AF488 | goat | 1:400 | Jackson Immuno Research | 111-545-045 | AB_2338049 | |
| Anti-rat | IgM  µ chain | Cy3 | goat | 1:600 | Jackson Immuno Research | 112-165-075 | AB_2338249 | |

**Table S3**: Primers used for RT-PCR and the generation of riboprobes (ISH)

| **Gene** | **Official Symbol**  **(Accession number)** | **Primer sequence (5'-3')** | **Annealing**  **temperature (˚C)** | **PCR**  **cycles** | **Product size**  **(bp)** | **reference** |
| --- | --- | --- | --- | --- | --- | --- |
| RPTPβ/ζ */ DSD-1-PG* (all isoforms) (PCR, ISH) | PTPRZ1 (NM_001081306.1) | actacctaacatgagttacg aagagtcatcggctcccgtat | 60°C | 32 | 640 | Garwood et al., 1999 |
| *DSD-1-PG* (PCR) | PTPRZ1 (NM_001081306.1) | tatgctaccccagaagcaca  tctgctggtggaccagaatt | 60°C | 32 | 400 | Garwood et al., 1999 |
| *C4ST-1* (PCR/ISH) | Chst11 (AB030378) | tgctggaagtgatgaggatg  ggtggttgatctctgggatg | 60 | 29 | 510 | Akita et al., 2008 |
| *C4ST-2* (PCR) | Chst12  (AJ289132) | cggctctcatgatccttttg  tcatcactcgcttccagttg | 60 | 32 | 519 | Akita et al., 2008 |
| *C4ST-3* (PCR)  (XM_355798) | Chst13  (NM_027928.1) | atgggaagacgctcctgttg  gcacgaagagaaaggtcaggtag | 60 | 38 | 505 | Akita et al., 2008 |
| *GalNAc4S-6ST* (PCR) | Chst15  (AB187269) | ttgttggtatgaggagttctcg  aggcatggatgaagtcttgg | 60 | 33 | 548 | Akita et al., 2008 |
| *C6ST-1* (PCR) | Chst3  (NM_016803) | aggcagatacgtcttgttcctg  agcacatacaggtcgcatagc | 60 | 31 | 528 | Akita et al., 2008 |
| *C6ST-1* (ISH) | Chst3  (NM_016803) | gggcaagtatgagaactggaag  agacatcccccactacgtga | 60 | 32 | 505 | Akita et al., 2008 |
| *C6ST-2* (PCR/ISH) | Chst7  (AB046929) | cttcttgtcccctctgtactgg  gagcagatgaccttgttggtc | 60 | 32 | 527 | Akita et al., 2008 |
| *UA2OST* (PCR) | Ust  (NM_177387) | gatgaagaagaagcagcagcag  acctggagaagttgaggaagtg | 65 | 35 | 533 | Akita et al., 2008 |
| *α-ACTIN* (PCR) | Actb  (NM_007393) | tatgccaacacagtgctgtctggtgg  agaagcacttgcggtgcacgatgg | 60 | 25 | 246 | Akita et al., 2008 |

**Movie legends (253 words)**

**Movie M1: Time-lapse video of control cells in the presence of EGF.**

The video shows a sequence of phase contrast images acquired over 90 hours. As the cells are proliferative, the density of the culture strongly increased from the beginning to the end of the video. The cultured spinal cord NSPCs were imaged every 5 minutes to achieve this movie.

**Movie M2: Time-lapse video of control cells in the presence of FGF2.**

The video shows a sequence of 90 hours. The cultured spinal cord NSPCs were imaged every 5 minutes. Please note that the cells in this condition displayed a different morphology compared to the EGF treated culture.

**Movie M3: Time-lapse video and exemplary lineage tree of sodium chlorate treated cells in the presence of EGF.**

The video shows the sodium chlorate treated cells on the right in phase contrast, while the lineage tree of an exemplary mother cell and its progeny (marked with red numbers) unfolds on the left side image by image.

**Movie M4: Time-lapse video and exemplary lineage tree of sodium chlorate treated cells in the presence of FGF2.**

The video shows the sodium chlorate treated cells on the right in phase contrast, while the lineage tree of an exemplary mother cell and its progeny (marked with red numbers) unfolds on the left image by image. The density of the culture was reduced at the end of the video in comparison to the chlorate treated cells in the presence of EGF, which underlines its impact on FGF2-dependent cell cycle progression.
