# Supplementary material for: Sulfation of Glycosaminoglycans Modulates the Cell Cycle of Embryonic Mouse Spinal Cord Neural Stem Cells
Source: Front Cell Dev Biol. 2021 Jun 8;9:643060. doi: 10.3389/fcell.2021.643060 (PMC8217649; doi:10.3389/fcell.2021.643060)
Supplement: Supplementary file 2 [file Table_2.docx]

Table S2: Primary and secondary antibodies used for immunohistochemistry

| **antigen** | **antibody (clone)** | **type** | **species** | **dilution IHC** | **reference/ manufacturer** | **order number** | **RRID** |
| --- | --- | --- | --- | --- | --- | --- | --- |
| DSD-1-PG/ RPTPβ/ζ | KAF13[2] | pAB | rabbit | 1:300 | Faissner 1994 |  |  |
| 473HD epitope | 473HD | IgM | rat | 1:300 | Faissner 1988 |  |  |
| βIII-tubulin | α−βIII-tubulin (SDL.3D10) | IgG2b | mouse | 1:300 | Sigma | T8660 | AB_477590 |
| Islet-1/2 | 39.4D5 | IgG2b | mouse | 1:200 | Developmental Studies Hybridoma Bank (DSHB), Jessell, T.M. / Brenner-Morton, S. | 39.4D5 | AB_2314683 |

| **antibody** | **type** | **conjugate** | **host** | **Dilution IHC** | **manufacturer** | **order number** | **RRID** |
| --- | --- | --- | --- | --- | --- | --- | --- |
| anti-rabbit | IgG | AF488 | goat | 1:400 | Jackson immuno Research | 111-545-045 | AB_2338049 |
| anti-rat | IgM  µ chain | Cy3 | goat | 1:600 | Jackson immuno Research | 112-165-075 | AB_2338249 |
