# Supplementary material for: Sulfation of Glycosaminoglycans Modulates the Cell Cycle of Embryonic Mouse Spinal Cord Neural Stem Cells
Source: Front Cell Dev Biol. 2021 Jun 8;9:643060. doi: 10.3389/fcell.2021.643060 (PMC8217649; doi:10.3389/fcell.2021.643060)
Supplement: Supplementary file 3 [file Table_3.docx]

Table S3: Primers used for RT-PCR and the generation of riboprobes (ISH)

| **Gene** | **Official Symbol**  **(Accession number)** | **Primer sequence (5'-3')** | **Annealing**  **temperature (˚C)** | **PCR**  **cycles** | **Product size**  **(bp)** | **reference** |
| --- | --- | --- | --- | --- | --- | --- |
| *RPTPβ/ζ / DSD-1-PG* (all isoforms) (PCR, ISH) | PTPRZ1 (NM_001081306.1) | actacctaacatgagttacg aagagtcatcggctcccgtat | 60°C | 32 | 640 | Garwood |
| *DSD-1-PG* (PCR) | PTPRZ1 (NM_001081306.1) | tatgctaccccagaagcaca  tctgctggtggaccagaatt | 60°C | 32 | 400 | Garwood |
| *Chst11* (PCR/ISH) | Chst11 (AB030378) | tgctggaagtgatgaggatg  ggtggttgatctctgggatg | 60°C | 29 | 510 | Akita |
| *Chst12* (PCR) | Chst12  (AJ289132) | cggctctcatgatccttttg  tcatcactcgcttccagttg | 60°C | 32 | 519 |  |
| *Chst13* (PCR) | Chst13  (NM_027928.1) | atgggaagacgctcctgttg  gcacgaagagaaaggtcaggtag | 60°C | 38 | 505 |  |
| *Chst15* (PCR) | Chst15  (AB187269) | ttgttggtatgaggagttctcg  aggcatggatgaagtcttgg | 60°C | 33 | 548 |  |
| *Chst3* (PCR) | Chst3  (NM_016803) | aggcagatacgtcttgttcctg  agcacatacaggtcgcatagc | 60°C | 31 | 528 |  |
| *Chst3* (ISH) | Chst3  (NM_016803) | gggcaagtatgagaactggaag  agacatcccccactacgtga | 60°C | 32 | 505 |  |
| *Chst7* (PCR/ISH) | Chst7  (AB046929) | cttcttgtcccctctgtactgg  gagcagatgaccttgttggtc | 60°C | 32 | 527 |  |
| *Ust* (PCR) | Ust  (NM_177387) | gatgaagaagaagcagcagcag  acctggagaagttgaggaagtg | 65°C | 35 | 533 |  |
| *β-ACTIN* (PCR) | Actb  (NM_007393) | tatgccaacacagtgctgtctggtgg  agaagcacttgcggtgcacgatgg | 60°C | 25 | 246 | Akita |
